# Joint Session of Ohio and Michigan State Veterinary Medical Association

**Published:** 1891-09

**Authors:** Wm. H. Gribble

**Affiliations:** Secretary. Washington, C. H. Ohio.


### SOCIETY PROCEEDINGS.

*Joint Session of Ohio and Michigan State Veterinary Medical Associations.* Held at Abstract Hall, Detroit, Michigan, July 22, 1891.

The meeting was called to order by Dr. Hawkin's, of Detroit, who in a few well chosen remarks stated the objects of the meeting, and moved that Dr. A. A. Grange, of Lansing, act as chairman. The motion was duly seconded, and the choice made unanimous. Dr. Grange thanked the members for the honor conferred, and stated that they must first proceed to elect a secretary. It was moved and supported that Dr. Gribble, Secretary of the Ohio Association, act as secretary. Carried.

There were present :

Drs. A. A. Grange, Lansing, Michigan; Jos. Hawkins, Detroit; J. J. Joy, S. Brenton, and Jas. Fleming, Detroit; Dr. W. J. Bracken, Fenton; Dr. W. B. Austin, Milford; Dr. J. W. Ferguson, Bay City; Dr. J. C. Whitney, Hillsdale ; Dr. Geo. Dumphy, Quincy ; Dr. Dell, Ann Arbor ; Dr. Thaborne, Lansing ; Dr. Smith, Adrain ; Dr. A. J. Thompson, Terre Haute, Ind.; Dr. J. W. Wilson, London, Ont.; Dr. J. W. Gibbs, St. Mary's, Ont.; Dr. J. C. Meyers, Cincinnati, Ohio; Dr. J. V. Newton, Toledo; Dr. W. R. Howe, Dayton; Dr. W. Shaw, Dayton; Dr. F.B.Hillock, Columbus; Dr. T. B. Cotton, Columbus; Dr. W. E. Wight, Delaware; Dr. J. Charlesworth, Springfield; Dr. E. R. Barnett, Akron; Dr. C. Christman, Akron; Dr. J. E. Campbell, Alliance; and Dr. Wm. H. Gribble, Washington, C. H.

Communications were read from several members of both Associations expressing regrets at inability to be present.

A communication was read from Prof. Liautard, asking for all papers, proceedings, discussions, etc., for publication in "American Veterinary Review."

Parke, Davis & Co., offered the meeting the use of one of their stenographers, and also invited members to visit their laboratories, etc., at 8 A. M., to-morrow morning. Carriages would be in waiting at the door of this building. Accepted.

Questions received from Dr. Meyers, of Cincinnati, were now read by the secretary and well discussed by the members present. The questions were :

1st. Has any one present had any practical observation as to whether Monorchide's or Cryptorchide's have the power of reproduction? 2nd. Have any of the gentlemen present ever examined the spermatic fluid of such animals and found spermatozoa?

In the discussion the opinion was universal that undeveloped testicles had no power to reproduce.

After closing the discussion of these foregoing questions, Dr. Gribble, of Washington, C. H. O., read a description of four cases of monstrosaties, two of foals and two of calves, all of which had been sired by the Dams own Sire.

In the discussion that followed the members did not believe that the breeding had anything to do with the cases described. A poor way to breed, but such cases were purely accidental was the opinion expressed. Next followed a discussion on the use of so-called impregnators. Many members present had used them, but found no good from their use, the universal opinion being, that they were the greatest humbugs ever perpetrated upon horse-owners.

Adjourned to meet at 8 A. M., to visit the manufactories of Parke, Davis & Co.

Wednesday, July 23d., met pursuant to adjournment and taking carriages in waiting visited the laboratories as per invitation, and all were well repaid for their visit.

Mr. S. S. Davis tendered the Association the use of his private yacht to visit the breeding farm, Clareview, thirteen miles up the Detroit river. Invitation accepted. Yacht to be at foot of Third St., at 1 P. M.

We now returned to Abstract Hall, meeting called to order at 10 A. M., with Dr. Grange in the chair, and Secretary W. H. Gribble.

Dr. Shaw made formal report of the death of Dr. Broaddus, of Indiana, and the chair appointed Drs. Shaw, Brenton and Dell, to draft resolutions.

The following were offered by the Committee :

### OBITUARY.

W. J. Broaddus, V. S., who died July 11, 1891, from drowning while bathing, was a young Veterinary Surgeon held in great esteem in the town of Connersville, Ind., where he practiced his profession. He was on several occasions the recipient of high marks of respect from the citizens of his town.

Dr. Broaddus was born in Connersville, Ind., in 1865, was educated in the schools of that place, and later entered the University of Indiana, where he took the usual course, including lectures on Veterinary Science. He graduated from there in the spring of 1888, and the autumn of the same year matriculated at the Ontario Veterinary College.

At this institution he won several prizes and the medal for best examination in Anatomy, and graduated in March, 1890, second in a class of two hundred. He returned to his native place and practiced his profession. He was noted for his pleasing address and his devotion to Veterinary Science.

Although he has only been in active practice a short time, his loss will be greatly felt by the citizens of Connersville.

*Whereas,* In the death of Dr. Broaddus it has pleased Almighty God to remove from our midst a worthy and esteemed friend, and

*Whereas,* The intimate relations and business intercourse with him have been most pleasant to the members of this association, and the Veterinary Profession it makes it befitting that we publicly record our appreciation of him ;

*Therefore Resolved,* That in the loss of Dr. Broaddus we lose a friend and valued member of our profession, and

*Resolved,* That with deep sympathy with the afflicted relations and friends of the deceased, we express our earnest hope, that even so great a bereavement may be over-ruled for their highest good, and

*Resolved,* That a copy of these resolutions be sent to his relatives, and also to the Veterinary Journals.

*Committee.*

Walter Shaw, Dayton, Ohio.
S. Brenton, Detroit, Mich.
E. Dell, Ann Arbor.

The report was accepted and ordered spread upon the minutes.

An essay on Digestion was read by the Secretary, which had been prepared by Dr. Logan, of Bellefontaine, Ohio. Owing to absence of essayist and short time at our disposal, discussion on his paper was postponed.

Next followed a paper by Dr. A. A. Grange, on the Horse. This was an excellent and highly interesting paper, combining as it did the evolution of the Equine, up to the present specimen.

Dr. Grange was asked to prepare his paper for publication, and furnish it to the Secretary of the meeting for that purpose.

After votes of thanks to those who had assisted us in enjoying the time spent, the meeting adjourned *sine die*, all members who desired, to meet at foot of Third St., at 1 P. M.

Washington, C. H. Ohio. WM. H. GRIBBLE, D. V. S.
*Secretary.*

